# A Genetic Variant rs1801274 in *FCGR2A* as a Potential Risk Marker for Kawasaki Disease: A Case-Control Study and Meta-Analysis

**DOI:** 10.1371/journal.pone.0103329

**Published:** 2014-08-05

**Authors:** Jiayu Duan, Jiao Lou, Qing Zhang, Juntao Ke, Yanqi Qi, Na Shen, Beibei Zhu, Rong Zhong, Zhenling Wang, Lifeng Liu, Jing Wu, Wei Wang, Fangqi Gong, Xiaoping Miao

**Affiliations:** 1 Department of Epidemiology and Biostatistics and State Key Laboratory of Environment Health, Ministry of Education Key Laboratory of Environment and Health, Ministry of Environmental Protection Key Laboratory of Environment and Health, School of Public Health, Tongji Medical College, Huazhong University of Science and Technology, Wuhan, Hubei, China; 2 Children's Hospital, Zhejiang University School of Medicine, Hangzhou, Zhejiang, China; Morehouse School of Medicine, United States of America

## Abstract

**Objectives:**

Recent genome-wide association study found rs1801274, a functional single nucleotide polymorphism (SNP) in IgG receptor gene *FCGR2A*, was associated with increased risk of Kawasaki disease (KD). However, subsequent studies on the role of this SNP were limited and controversial.

**Methods:**

A case-control study was conducted in a Chinese Han population including 428 KD patients and 493 controls to examine the association between rs1801274 and KD susceptibility. A meta-analysis was performed in combination with the relevant published studies to further clarify such an association.

**Results:**

Our case-control study found that rs1801274 was significantly associated with increased risk of KD in the Chinese Han population, with an odds ratio (OR) of 1.58 (95% CI = 0.96–2.62) for the GA genotype and 1.93 (95% CI = 1.16–3.19) for the AA genotype compared with the GG genotype. The result of meta-analysis further demonstrated that the A allele of rs1801274 was significantly correlated with KD risk under the allelic model (OR = 1.35, 95% CI = 1.27–1.44) without heterogeneity by fixed-effects model analysis (*Q* = 17.30, *p* = 0.139). Moreover, sensitivity analysis supported the robustness of this meta-analysis.

**Conclusion:**

These results further confirm that rs1801274 in the *FCGR2A* gene is significantly associated with increased risk of KD.

## Introduction

Kawasaki disease (KD, OMIM 611775), first reported by a Japanese pediatric doctor Tomisaku Kawasaki [1], is an acute, multi-systemic vasculitis that specifically attacks children under 5 years old. Currently, KD has become one of the leading causes of acquired heart disease of children in developed countries [2]–[5]. Clinical manifestations of KD include fever at least 5 days, bilateral conjunctival congestion, polymorphous rashes, cervical lymphadenopathy, changes of extremities and mucous membranes of upper respiratory tract [6]. Diagnosis of KD is mostly based on clinical criteria due to absence of specific tests and pathogen markers [7]. About 50% of KD patients suffer from heart damages, and a quarter of untreated patients finally develop coronary artery lesions (CALs), including coronary artery dilatation, coronary fistula and even myocardial infarction [8]–[10]. So far, high dose intravenous immunoglobulin (IVIG) in combination with oral aspirin is the only evidence-based therapy for KD, which could decrease the occurrence of coronary artery complications to 5–16% [11], [12]. It is reported that the annual incidence of KD is 69/100 000 in Taiwan, the third highest worldwide, and 30.7/100000 in mainland China [3], which is on the increase in recent years.

So far, the etiology of KD remains unclear even clinical features indicates an infectious agent may be involved in the pathogenesis of KD. Evidence from epidemiological studies highly suggested that genetic factors play an important role in this process. First, twins and siblings of affected children have a risk of KD 10 times higher than general population [13]. Second, the risk of KD is doubled in the children when their parents had a history of KD [14]–[16]. At last, the incidence of KD is 10 to 20 times higher in North-East Asian populations including Japanese and Koreans than that reported in Caucasians [17], [18]. To date, genome-wide association study (GWAS) and linkage analysis of KD have identified 13 genetic loci, which are associated with KD or CALs in Japanese, Europeans, Koreans and Taiwanese [19]–[24], including IgG receptor gene *FCGR2A* (Fc gamma receptor IIa). The single nucleotide polymorphism (SNP) rs1801274 in *FCGR2A* gene encodes an H131R substitution, which may be related to the development of KD.

*FCGR2A* is commonly expressed on immune responsive cells like macrophages, neutrophils, monocytes and dendritic cells [25] to boost phagocytosis and production of inflammatory mediators [26]. The A allele of rs1801274 results in a point mutation from arginine to histidine, leading to increased immune responses. In a variety of human immune system diseases, such as systemic lupus erythematosus [27], arthritis [28] and ulcerative colitis [29], aberrant expression or allelic variants of *FCGR2A* have been identified.

Given its genetic function, *FCGR2A* gene was assumed to be a potential marker of KD risk. However, the results from previous studies were inconsistent [22], [23], [37]–[40]. Hence, we conducted a case-control study to examine the association between *FCGR2A* gene and KD risk in a Chinese Han population from Zhejiang Province in Southeast China. Additionally, a meta-analysis was performed by combining the data of this study with the results from other relevant, previously published case-control studies, and the data of transmission/disequilibrium test (TDT) to further confirm such an association.

## Materials and Methods

### Study population

In this present study, a total of 428 KD patients were recruited from April 2009 to September 2012 in Children's Hospital, Zhejiang University in China. All the patients were unrelated children of Chinese Han ethnic origin. The diagnosis of KD was based on the latest diagnostic criteria released by the Japan Kawasaki Disease Research Committee in 2002 [7]. The controls were 493 gender-matched unrelated healthy Chinese children of Han ethnic origin. They were randomly selected from the children taking physical examination in Children's Hospital, Zhejiang University for admission to primary school. All patients were subjected to two-dimensional echocardiography during febrile episode and after hospital discharge. And 414 patients received IVIG at daily dose of 1 g/kg for 2 days or a single infusion of 2 g/kg and oral aspirin at daily dose of 80 mg/kg. CAL was defined as coronary arteries with a diameter (inner border to inner border) ≥3 mm in children younger than 5 years old, >4 mm in children above 5 years, or the diameter was >1.5 times wider than that of the adjacent vessel [30], [31].

This study was approved by the Ethics Committee of Children's Hospital, Zhejiang University School of Medicine, and Huazhong University of Science and Technology (HUST). Written informed consent forms were obtained from parents/guardians of all the participants.

### SNP Genotyping

Genomic DNA was extracted from 2 ml peripheral blood sample with the RelaxGene Blood DNA System DP319-02 (Tiangen, Beijing, China) following the manufacturer's instructions. TaqMan SNP Genotyping Assay was used to determine rs1801274 genotypes using a 7900 HT Fast Real-Time PCR System (Applied Biosystems, Foster City, CA).

### Statistical analysis

The gender distribution and genotypic frequencies were estimated by Pearson χ^2^ test between patients and controls. Hardy-Weinberg equilibrium (HWE) was tested by a goodness-of-fit χ^2^ test for the distribution of genotypic frequencies among controls. After adjustment of gender, unconditional logistic regression analyses were performed to calculate the odds ratios (ORs) and 95% confidence intervals (CIs) for the effect of the SNP on KD and CALs in patients and controls, as well as the patients with and without CALs, respectively. The analysis was conducted under dominant, recessive and additive models respectively to avoid the bias of genetic models. All the statistical analyses were performed with SPSS 11.0 software (SPSS, Inc., Chicago, IL, USA). Power calculation was carried out using Power version 3.0 with the current sample size and genotype frequency. All *p* values were two-tailed. *P*<0.05 was considered statistically significant.

### Meta-analysis for the association between rs1801274 and KD risk

Several databases including EMBASE, PubMed, and ISI Web of Science were searched up to August 28, 2013 to identify the relevant case-control and TDT studies on the association between rs1801274 and KD. The search terms *rs1801274*, *Fc gamma receptor*, *polymorphism*, and *Kawasaki disease* were used without language restriction. References cited in the retrieved articles, relevant studies or review articles on this topic were also included. The selected studies should meet the following criteria: (1) a case-control or family-based TDT study assessing the association between rs1801274 and KD; (2) containing integrated information about genotype or allele frequency for risk estimates, or original data; (3) genotypes in control groups were fit to Hardy-Weinberg equilibrium (*p*>0.05). Animal studies, commentaries and case reports were excluded. If subjects were overlapped in several studies, only the one with more complete design or larger sample size was selected.

The information extracted from each eligible article included the name of first author, year of publication, study group, ethnicity, method of genotyping, design type and diagnostic criteria for KD.

Cochran's χ^2^-based *Q* statistic test was used to estimate between-study heterogeneity. It was considered statistically significant when *p*<0.1. In this case, a random-effects model, using DerSimonian and Laird method, was further applied to calculate the pooled OR. Otherwise, a fixed-effects model, using Mantel-Haenszel method, was employed. In order to integrate the results from both case-control and TDT studies, the method described by Kazeem and Farrall [32] was referred. Estimate of combined OR and its standard error (SE) was calculated by a weighted analysis method [33]. All the evaluations were conducted using Catmap software, which could be downloaded from http://www.r-project.org [34]. In addition, sensitivity analysis was used to assess the influence of each study on the overall estimate after removal of this study [35]. Publication bias was assessed by funnel plot and regression test. All *p* values were from two-tailed test with a significant level at 0.05 except those for heterogeneity. The meta-analysis was carried out using Catmap software V1.6.

## Results

### Characteristics of study population

A total of 428 KD patients and 493 healthy controls were included in this case-control study. Characteristics of all the patients and controls were summarized in Table 1. Males accounted for 61.45% of te ptients and 61.46% of the controls (χ^2^ = 0.000, *p* = 0.997). Generally, CALs were detected in 29 patients. The statistical power was 0.846 for the current sample size (493/428), and 0.190 for CAL group (399/29).

**Table 1:** The characteristics of patients with Kawasaki disease and healthy controls in current study.

Variables	Case (n = 428)	Control (n = 493)	*P* value
Gender, N (%)			0.997^a^
Male	263 (61.45)	303 (61.46)	
Female	165 (38.55)	190 (38.54)	

a *P* value was calculated by χ^2^ test.

### Association analysis

The genotyping of SNP rs1801274 was successful in 99.35% of the study subjects (425/428 patients and 493/493 controls). The genotypic frequency distribution was in agreement with HWE (*p* = 0.486) in the controls. As shown in Table 2, rs1801274 was significantly associated with increased risk of KD in those carrying the A allele or AA genotype compared to those carrying the G allele or GG genotype (A versus G: OR = 1.29, 95% CI = 1.06–1.58; AA versus GG: OR = 1.93, 95% CI = 1.16–3.19). Similarly, such a significant association was also demonstrated in dominant model (OR = 1.75, 95% CI = 1.08–2.84), recessive model (OR = 1.31, 95% CI = 1.01–1.70), or additive model (OR = 1.31, 95% CI = 1.07–1.61).

**Table 2:** Polymorphism of rs1801274 of the *FCGR2A* gene in the KD patients and healthy controls in current study.

Frequency	KD cases (%)	Controls (%)	OR	95% CI	*P* a
Genotype					
GG	27 (6.37)	52 (10.59)	Reference	Reference	1
GA	185 (43.63)	226 (46.03)	1.58	0.96–2.62	0.075
AA	212 (50.00)	213 (43.38)	1.93	1.16–3.19	**0.011** b
Allelic model	—	—	1.29	1.06–1.58	**0.012** b
Dominant model	—	—	1.75	1.08–2.84	**0.024** b
Recessive model	—	—	1.31	1.01–1.70	**0.045** b
Additive model	—	—	1.31	1.01–1.61	**0.010** b

a Adjusted the effect of gender and age. KD, Kawasaki disease.

b The values in bold indicate statistically significant (*p*<0.05).

The KD patients were then stratified into two groups based on development of CAL (Table S1 in File S1). Unexpectedly, no significant association was observed between the polymorphism and CAL formation in terms of genotype (A versus G: OR = 1.03, 95% CI = 0.57–1.87), dominant model (OR = 0.47 95% CI = 0.06–3.63), recessive model (OR = 1.08, 95% CI = 0.51–2.29), or additive model (OR = 1.04, 95% CI = 0.56–1.93).

### Relevant studies included in meta-analysis

**Figure 1:**
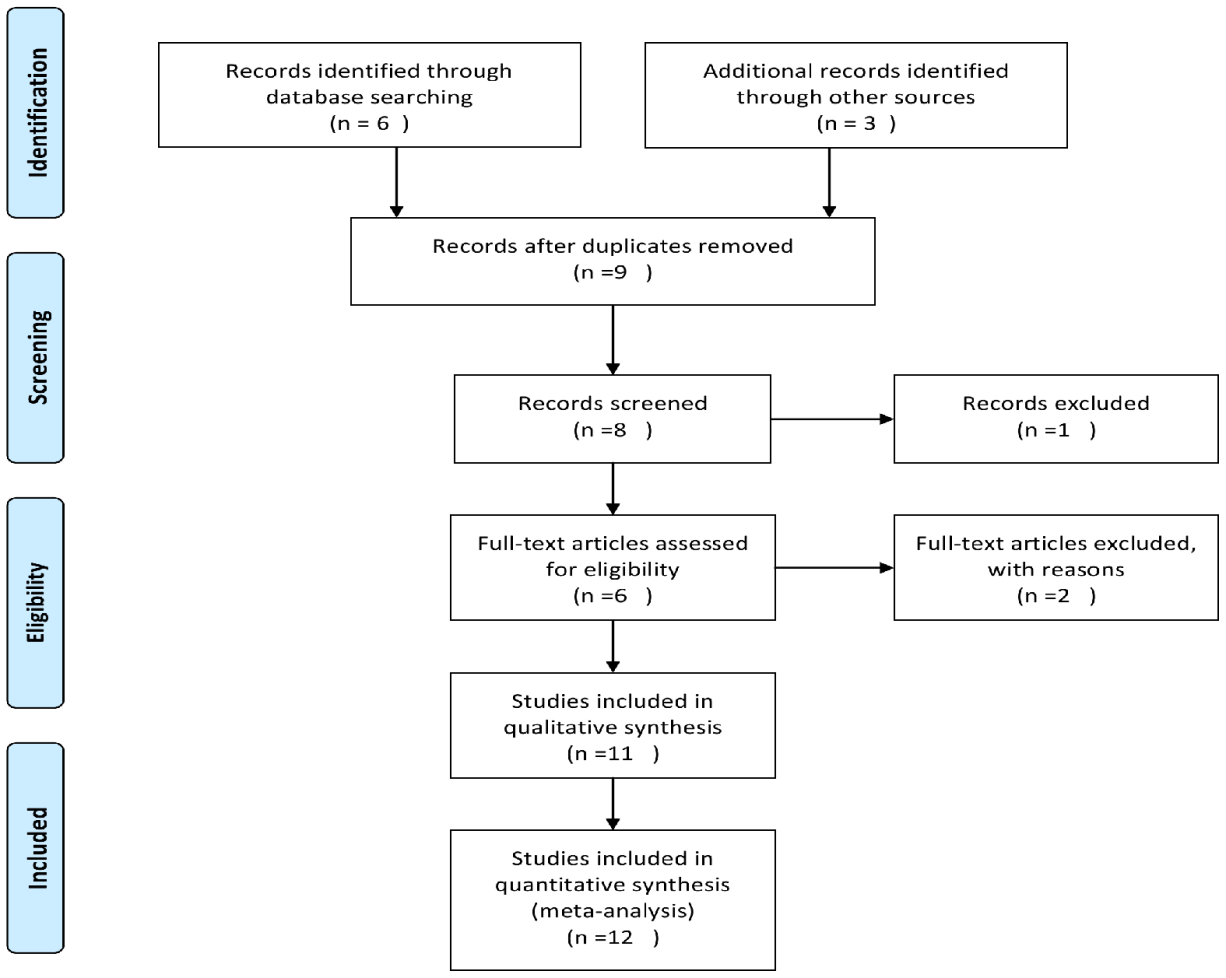
Flow chart of study selection.

After comprehensive search, nine potentially relevant articles were retrieved, of which one review and two relevant studies were excluded due to ineligibility (Figure 1). As a result, the meta-analysis finally contained twelve (including this present study) individual case-control studies and one TDT study [22], [23], [36]–[39], involving 3673 cases, 14226 controls and 586 families (Table 3).

**Table 3:** Characteristics of the studies on rs1801274 polymorphisms and risk of Kawasaki disease included in the meta-analysis.

First Author	Year	Country	Ethnicity	Study Method	Study Design	Genotyping	T/NT	Case/Control
ShoichiroTaniuchi [37]	2005	Japan	Asian	CC	Replication	RFLP		65/566
Maarten Biezeveld [38]	2006	Netherlands	Caucasian	CC	Replication	PCR-Direct sequencing		176/239
ChieaChuenKhor [22]	2011	Singapore	European	CC	GWAS	IllumiaBeadChip		405/6252
ChieaChuenKhor [22]	2011	Singapore	Asian	CC	Replication	SequenomMassARRAY		438/446
ChieaChuenKhor [22]	2011	Singapore	Asian	CC	Replication	Taqman-PCR		460/498
ChieaChuenKhor [22]	2011	Singapore	Asian	CC	Replication	Taqman-PCR		130/568
ChieaChuenKhor [22]	2011	Singapore	Mixed	TDT	Replication	SequenomMassARRAY	314/272	
Yoshihiro Onouchi [23]	2012	Japan	Asian	CC	GWAS	IlluminaBeadChip		428/3379
Yoshihiro Onouchi [23]	2012	Japan	Asian	CC	Replication	Invader Assay		470/378
Yoshihiro Onouchi [23]	2012	Japan	Asian	CC	Replication	Invader Assay		284/569
Yu Xiao Ji [39]	2013	China	Asian	CC	Replication	PCR-Direct sequencing		35/25
Yuanlong Yan [36]	2013	China	Asian	CC	Replication	Taqman-PCR		358/815
Current study	2013	China	Asian	CC	Replication	Taqman-PCR		428/493

Abbreviations: CC, Case Control; TDT, transmission/disequilibrium test; T, transmission; NT, non-transmission.

### Overall meta-analysis of the association between rs1801274 and KD risk

**Figure 2:**
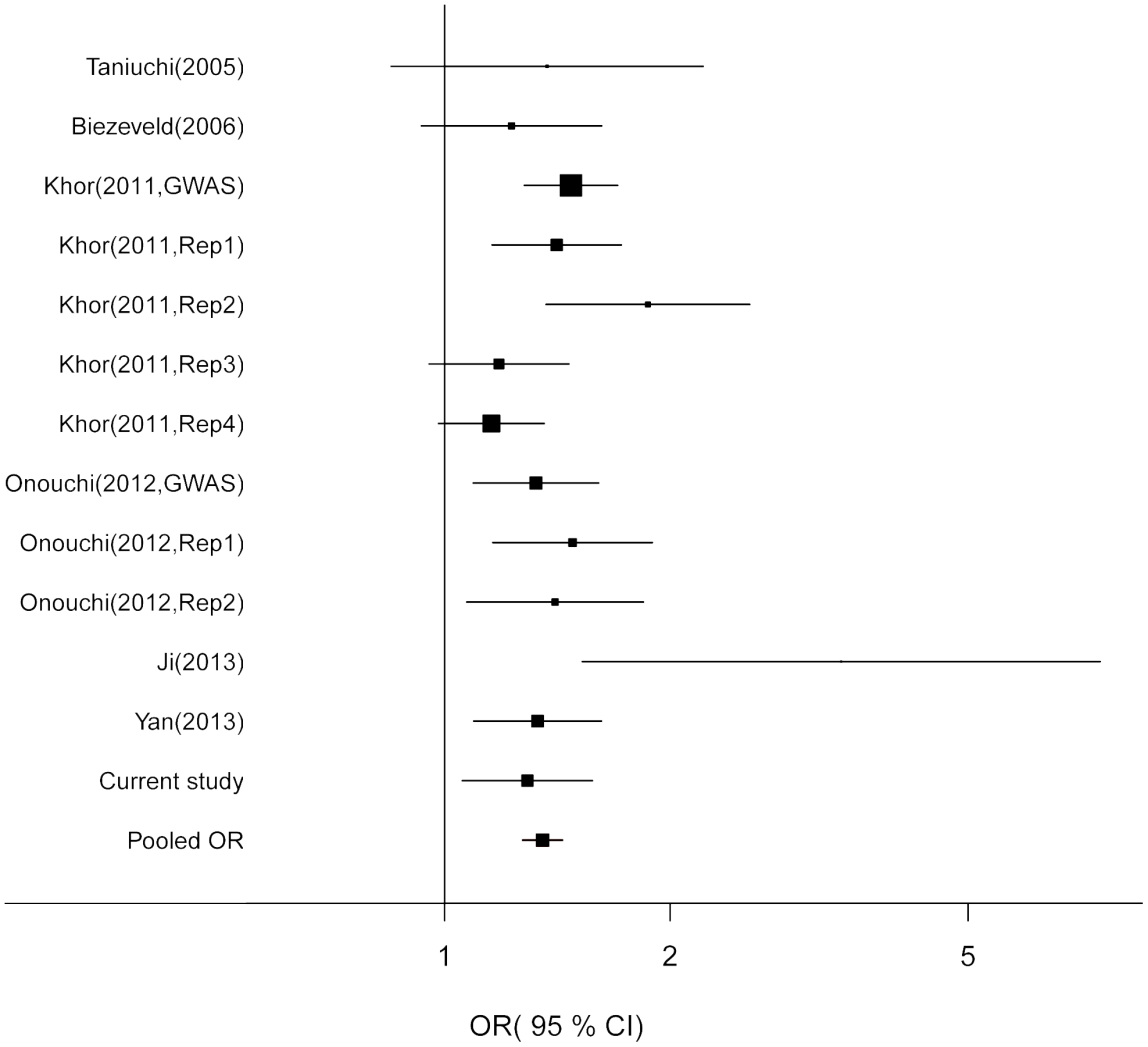
The forest plot for the association between the A allelic variant and the risk of Kawasaki disease. For each study, the estimate of OR and its 95% CI is plotted with a box and a horizontal line. Fixed-effects pooled OR = 1.35, 95% CI = 1.27–1.44, *p*<0.001; *Q* = 17.30, *P*_heterogeneity_ = 0.139.

No evidence of significant heterogeneity was detected (*Q* = 17.30, *p* = 0.139). Therefore, a fixed-effects model was employed to calculate the pooled OR (Table 4). Compared with the G allele of rs1801274, the A allele showed a pooled OR of 1.35 (95% CI = 1.27–1.44). The forest plot was shown in Figure 2.

**Table 4:** *FCGR2A* rs1801274 allele A and the risk of Kawasaki disease in meta-analysis.

Study ID	A/G in cases	A/G in controls	Transmitted A	Non-transmitted A	OR (95% CI)
Taniuchi (2005) [37]	108/22	885/247	—	—	1.37 (0.85–2.21)
Biezeveld (2006) [38]	202/150	250/228	—	—	1.23 (0.93–1.62)
Khor (2011) [22]	616/260	559/333	—	—	1.41 (1.16–1.72)
Khor (2011) [22]	200/60	728/408	—	—	1.87 (1.37–2.55)
Khor (2011) [22]	726/194	757/239	—	—	1.18 (0.95–1.46)
Khor (2011) [22]	459/351	5877/6627	—	—	1.47 (1.28–1.70)
Khor (2011) [22]	—	—	314	272	1.15 (0.98–1.36)
Onouchi (2012) [23]	720/136	5406/1352	—	—	1.32 (1.09–1.61)
Onouchi (2012) [23]	790/150	590/166	—	—	1.48 (1.16–1.89)
Onouchi (2012) [23]	482/86	910/228	—	—	1.40 (1.07–1.84)
Ji (2013) [39]	55/15	26/24	—	—	3.38 (1.53–7.50)
Yan (2013) [36]	528/188	1106/524	—	—	1.33 (1.09–1.62)
Current study (2013)	609/239	652/330	—	—	1.29 (1.06–1.57)
Pooled OR	—	—	—	—	1.35 (1.27–1.44)^a^

a Fixed-effects OR, *Q* = 17.30, *P*_heterogeneity_ = 0.139, *p*<0.001.

### Subgroup analysis

The epidemiological characteristics of KD have shown that the risk of KD in Asian children is much higher than that in the children of other ethnic origin. So, Asian children were treated as a subgroup to further explore the susceptibility of KD in this single ethnicity. The pooled OR indicated that the A allele was still associated with a higher risk of KD in Asian children compared with the G allele (OR = 1.37, 95% CI = 1.27–1.48). No significant heterogeneity was detected under the fixed-effects model (*Q* = 11.61, *p* = 0.236, Table S2 in File S1).

### Sensitivity analysis

To evaluate the effect of each single study on the pooled estimate, we performed a sensitivity analysis by removing one study each time in turn. It was found that all the results hardly changed after removal of each study, suggesting the robustness of our results (Table S3 in File S1).

### Cumulative meta-analysis

Cumulative meta-analysis was also conducted in allelic model by gradually including each additional study according to chronological order. The 95% CI for pooled OR became progressively narrower after adding one more study (Figure S1 in File S1). It indicated that the precision of our estimation was gradually improved after adding more samples.

### Publication bias evaluation

**Figure 3:**
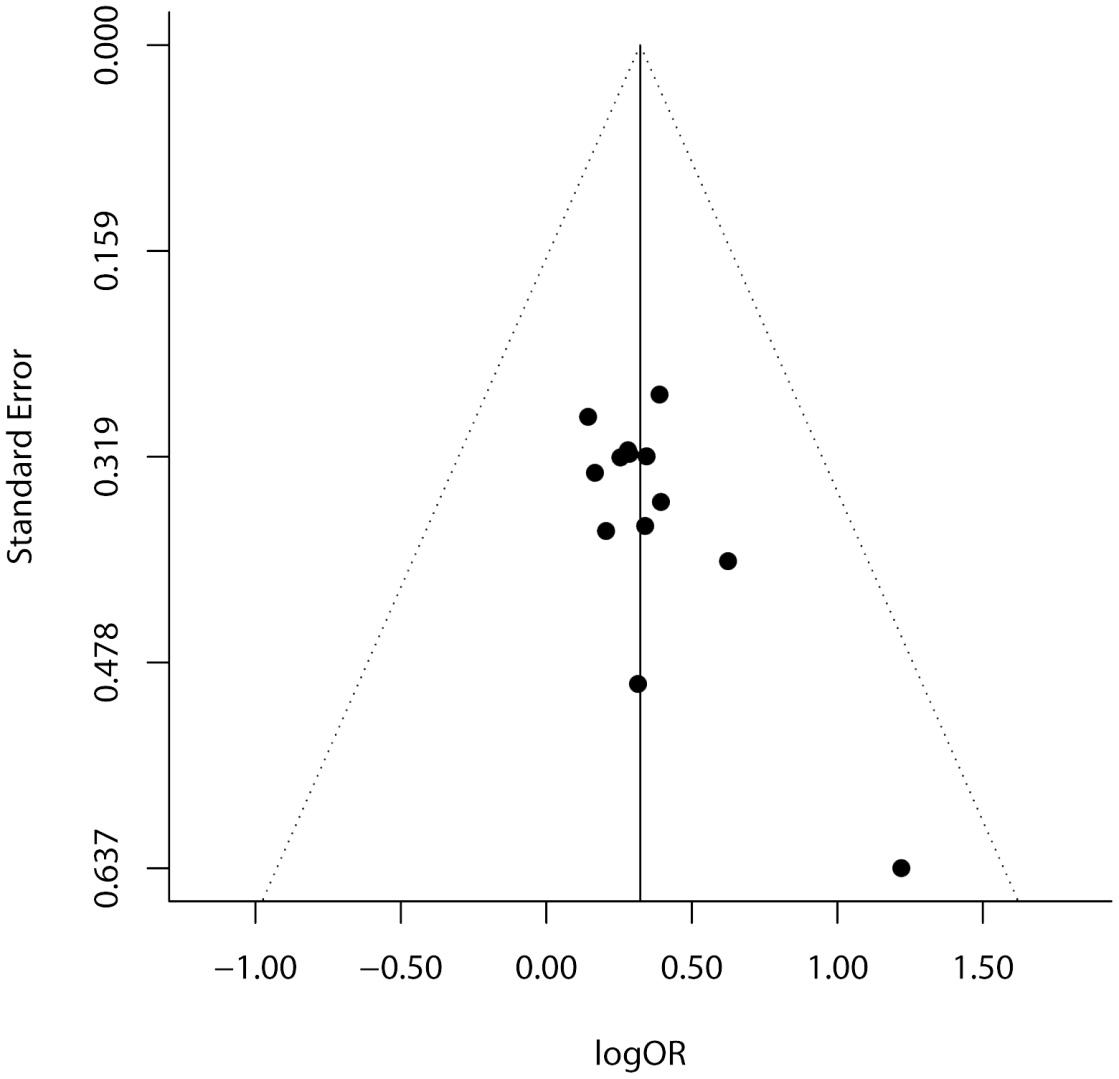
The funnel plot of natural logarithm of OR against inverse standard error in each study. Regression test for funnel plot asymmetry *p* = 0.224.

Publication bias was examined by funnel plot (Figure 3) and regression test. Dots in the funnel plot were mostly symmetrically distributed. The regression test further confirmed that there was no publication bias in this meta-analysis (*p* = 0.225).

## Discussion

In this present study, the individuals carrying the A allele or AA genotype of rs1801274 were at higher risk of developing KD but were not associated with CAL formation significantly. Subsequent meta-analysis further supported the association between this SNP with increased KD risk. Cumulative and sensitivity analyses confirmed the reliability and robustness of our results, and with little publication bias.

These findings are biologically plausible for the following reasons. First, the original GWAS on KD conducted by Khor et al [22] revealed this risk association in multi-ethnic populations and further tested this locus in four independent studies with different ethnic origins, including three case-control panels (Taiwanese, Korean and Chinese) and one TDT (non-European). Second, KD is an immune-mediated multi-systemic vasculitis and may have an unknown infectious trigger in addition to genetically susceptible factors. One function of FCGRs is to regulate the interaction between phagocytes and IgG antibodies. Functional assays demonstrated that this variant (G>A transition) enhanced *FCGR2A*'s binding affinity to IgG indirectly, resulting in activation of *FCGR2A* signaling pathway and up-regulation of IgG2-opsonized phagocytosis [40], [41]. Excessive inflammatory responses can produce adverse effect on healthy tissues such as extremity edema, hyperalgesia and swelling tongue, and further leading to clinic manifestations of KD.

Moreover, as the most severe complication of KD, CAL is the leading cause of long-term morbidity and mortality of KD patients. A recent study on endothelial function indicated that the G allele of rs1801274 was associated with endothelium-dependent vasodilatation and nitric oxide activity when endothelial cells were stimulated [42]. Another relevant study suggested that *FCGR2A* on the cells with AA genotype had stronger binding affinity to C-reactive protein (CRP), which showed similar functional activities as IgG2 [43]–[45] and FCGRs [46], [47]. Plasma CRP level increases significantly during acute phase of KD, which is an independent predictor for acute coronary syndromes [48]–[51]. These results imply that *FCGR2A* may affect the pathophysiology of coronary artery indirectly. However, the present case-control study in Chinese Han ethnic children did not confirm whether rs1801274 was associated with CAL. This may be due to the lack of enough CAL cases in our study.

Several limitations should be kept in mind when interpreting the results of this study. First, the sample size was relatively small, which might affect the power to detect the association between this SNP and CAL. Second, KD is a complex disease associated with both genetic and environmental factors. Local environmental data were not included in this analysis which limited the interpretation of these results and evaluation of the gene-environment interaction.

In conclusion, this present study has provided more evidence to support the concept that the functional polymorphism rs1801274 in *FCGR2A* is significantly associated with the increased risk of KD.

## Supporting Information

Figure S1 The forest plot for the cumulative meta-analysis. Fixed-effects pooled OR = 1.35, 95% CI = 1.27–1.44, *P*<0.001.
(TIF)

Checklist S1 PRISMA Checklist.
(DOC)

File S1 Table S1, Polymorphism of rs1801274 of the *FCGR2A* gene in patients with CALs and non-CALs in current study. Table S2, *FCGR2A* rs1801274 risk allele A in Meta analysis (Asian subgroup).Table S3, Sensitivity analysis of the allelic model.
(DOCX)
