# Supplementary figures and images for: A Genetic Variant rs1801274 in FCGR2A as a Potential Risk Marker for Kawasaki Disease: A Case-Control Study and Meta-Analysis
Source: PLoS One. 2014 Aug 5;9(8):e103329. doi: 10.1371/journal.pone.0103329 (PMC4122468; doi:10.1371/journal.pone.0103329)

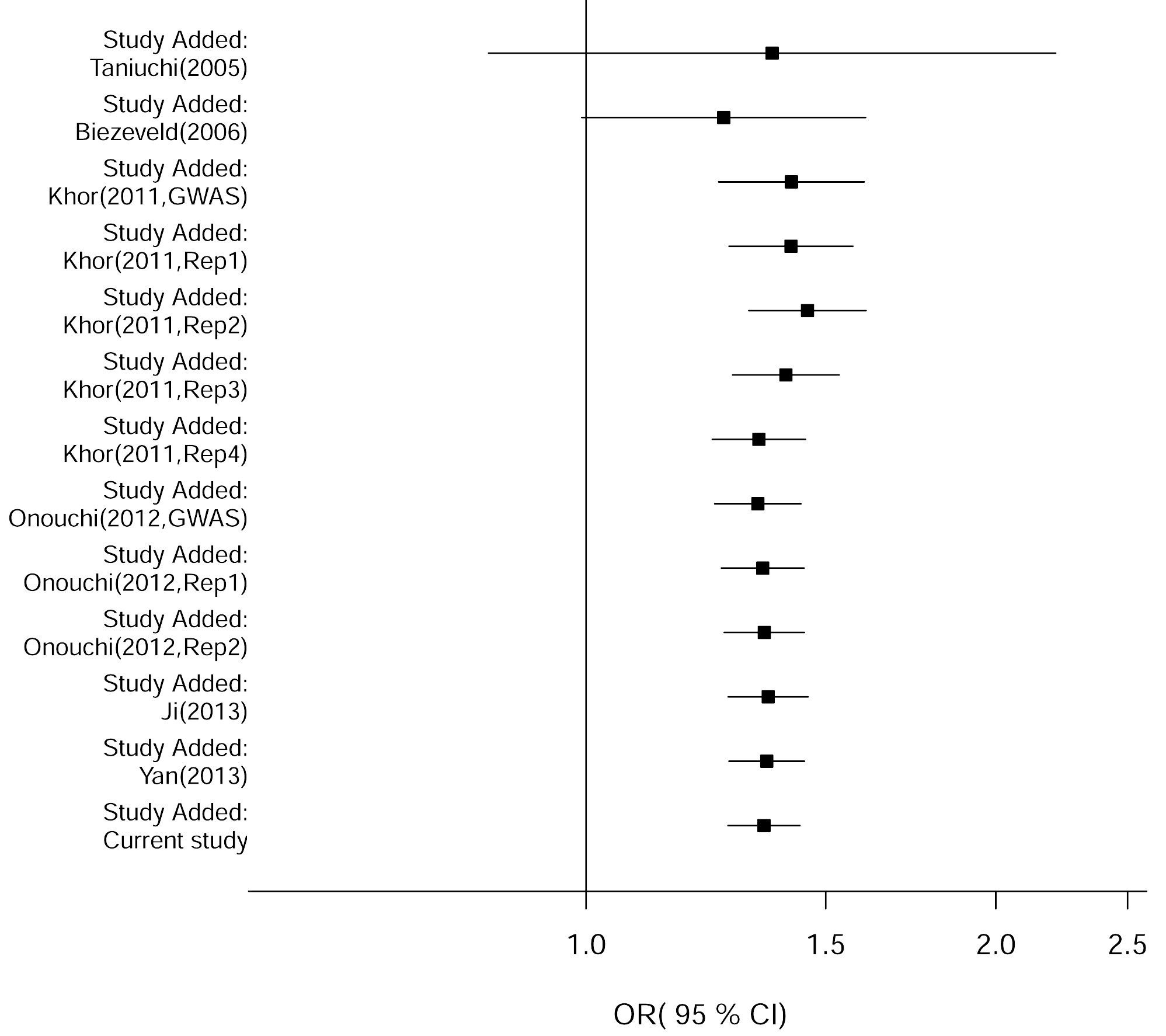

Supplement: Figure S1 — The forest plot for the cumulative meta-analysis. Fixed-effects pooled OR = 1.35, 95% CI = 1.27–1.44, P<0.001. (TIF) [file pone.0103329.s001.tif]
