# Supplementary material for: A Genetic Variant rs1801274 in FCGR2A as a Potential Risk Marker for Kawasaki Disease: A Case-Control Study and Meta-Analysis
Source: PLoS One. 2014 Aug 5;9(8):e103329. doi: 10.1371/journal.pone.0103329 (PMC4122468; doi:10.1371/journal.pone.0103329)
Supplement: File S1 — Table S1, Polymorphism of rs1801274 of the FCGR2A gene in patients with CALs and non-CALs in current study. Table S2, FCGR2A rs1801274 risk allele A in Meta analysis (Asian subgroup).Table S3, Sensitivity analysis of the allelic model. (DOCX) [file pone.0103329.s003.docx]

**Supplementary Table 1.** Polymorphism of rs1801274 of the *FCGR2A* gene in patients with CALs and non-CALs in current study

| Frequency | CALs(%) | non-CALs(%) | OR | 95% CI | *P*^a^ |
| --- | --- | --- | --- | --- | --- |
| Genotype |  |  |  |  |  |
| GG | 1 (3.44) | 26 (6.58) | reference | reference | 1 |
| GA | 14 (48.28) | 171 (43.29) | 2.13 | 0.27-16.87 | 0.474 |
| AA | 14 (48.28) | 198 (50.13) | 1.84 | 0.23-14.56 | 0.564 |
| Allelic model | -- | -- | 1.03 | 0.57-1.87 | 0.917 |
| Dominant model | -- | -- | 0.47 | 0.06-3.63 | 0.472 |
| Recessive model | -- | -- | 1.08 | 0.51-2.29 | 0.847 |
| Additive model | -- | -- | 1.04 | 0.56-1.93 | 0.913 |

^a^Adjusted the effect of gender and age.

**Supplementary Table 2.** *FCGR2A* rs1801274 risk allele A in Meta analysis (Asian subgroup)

| Study ID | A/G in cases | A/G in controls | Transmitted A | Non-transmitted A | OR (95% CI) |
| --- | --- | --- | --- | --- | --- |
| Taniuchi(2005) | 108/22 | 885/247 | -- | -- | 1.37 (0.85-2.21) |
| Khor(2011)  Khor(2011)  Khor(2011)  Onouchi(2012)  Onouchi(2012)  Onouchi(2012)  Ji(2013)  Yan(2013)  Current study(2013)  Pooled OR | 616/260  200/60  726/194  720/136  790/150  482/86  55/15  528/188  609/239  -- | 559/333  728/408  757/239  5406/1352  590/166  910/228  26/24  1106/524  652/330  -- | --  --  --  --  --  --  --  --  --  -- | --  --  --  --  --  --  --  --  --  -- | 1.41 (1.16-1.72)  1.87 (1.37-2.55)  1.18 (0.95-1.46)  1.32 (1.09-1.61)  1.48 (1.16-1.89)  1.40 (1.07-1.84)  3.38 (1.53-7.50)  1.33 (1.09-1.62)  1.29 (1.06-1.57)  1.37 (1.27-1.48)^*^ |

^*^ Fixed-effects OR, *Q*=11.61, P_heterogeneity_=0.236, *P* < 0.001

**Supplementary Table 3.** Sensitivity analysis of the allelic model

| Study omitted | OR (95%CI) | *P* | *Q* | *P*_heterogeneity_ |
| --- | --- | --- | --- | --- |
| Taniuchi(2005)  Biezeveld(2006)  Khor(2011)  Khor(2011)  Khor(2011)  Khor(2011)  Khor(2011)  Onouchi(2012)  Onouchi(2012)  Onouchi(2012)  Ji(2013)  Yan(2013)  Current study(2013) | 1.35 (1.27- 1.43)  1.35 (1.27-1.44)  1.34 (1.26-1.43)  1.33 (1.25-1.41)  1.36 (1.28-1.45)  1.32 (1.23-1.41)  1.38 (1.29-1.48)  1.35 (1.27-1.44)  1.34 (1.26-1.43)  1.34 (1.26-1.43)  1.34 (1.26-1.42)  1.35 (1.26-1.44)  1.35 (1.27-1.44) | <0.001  <0.001  <0.001  <0.001  <0.001  <0.001  <0.001  <0.001  <0.001  <0.001  <0.001  <0.001  <0.001 | 17.30  16.82  17.10  13.02  15.68  15.56  13.10  17.25  16.72  17.22  12.16  17.28  17.07 | 0.099  0.113  0.105  0.292  0.154  0.158  0.287  0.101  0.116  0.102  0.351  0.100  0.106 |
